# Machine Learning and Graph Signal Processing Applied to Healthcare: A Review

**DOI:** 10.3390/bioengineering11070671

**Published:** 2024-07-02

**Authors:** Maria Alice Andrade Calazans, Felipe A. B. S. Ferreira, Fernando A. N. Santos, Francisco Madeiro, Juliano B. Lima

**Affiliations:** 1 Centro de Tecnologia e Geociências, Universidade Federal de Pernambuco, Recife 50670-901, Brazil; juliano.lima@ufpe.br; 2 Unidade Acadêmica do Cabo de Santo Agostinho, Universidade Federal Rural de Pernambuco, Cabo de Santo Agostinho 54518-430, Brazil; felipe.bsferreira@ufrpe.br; 3 Institute for Advanced Studies, Universiteit van Amsterdam, 1012 WP Amsterdam, The Netherlands; f.a.nobregasantos@uva.nl; 4 Escola Politécnica de Pernambuco, Universidade de Pernambuco, Recife 50720-001, Brazil; madeiro@poli.br

**Keywords:** deep learning, graph signal processing, health, machine learning

## Abstract

Signal processing is a very useful field of study in the interpretation of signals in many everyday applications. In the case of applications with time-varying signals, one possibility is to consider them as graphs, so graph theory arises, which extends classical methods to the non-Euclidean domain. In addition, machine learning techniques have been widely used in pattern recognition activities in a wide variety of tasks, including health sciences. The objective of this work is to identify and analyze the papers in the literature that address the use of machine learning applied to graph signal processing in health sciences. A search was performed in four databases (Science Direct, IEEE Xplore, ACM, and MDPI), using search strings to identify papers that are in the scope of this review. Finally, 45 papers were included in the analysis, the first being published in 2015, which indicates an emerging area. Among the gaps found, we can mention the need for better clinical interpretability of the results obtained in the papers, that is not to restrict the results or conclusions simply to performance metrics. In addition, a possible research direction is the use of new transforms. It is also important to make new public datasets available that can be used to train the models.

## 1. Introduction

Graph signal processing (GSP) is an emerging research field, which focuses on generalizing the classical concepts of signal processing in order to expand them to graphs [1]. The need for GSP is related to the considerable amount of information that can be represented as a signal whose samples lie over irregular structures that can be modeled as graphs [1,2]. Among the GSP application scenarios that have attracted the attention of researchers and have been documented in recent studies, one can mention forecasting in the financial market [3], 3D point clouds [4], the Internet of Things (IoT) [5], traffic [6], and sensor, social, physical, and biological networks [7,8,9,10].

In the practical use of GSP, machine learning (ML) techniques and, in particular, deep learning (DL) techniques have been playing an important role. This is due to the fact that deep neural networks are adaptable to solving a wide range of problems, providing better or competitive results, when compared to other techniques. The extension of ML to non-Euclidean data gave rise to graph learning (GL) [11] and, consequently, to graph neural networks (GNNs). Such networks have also provided good results in several applications [12,13]. Regarding deep learning on graphs [11], specifically, we can mention the graph convolutional neural networks (GCNNs), in which deep networks with convolutional layers are proposed, such as the operations performed by the traditional convolutional neural network (CNN), but in this case, applied to problems in the non-Euclidean domain, i.e., in graphs [14,15,16].

Among the areas that have been highlighted in recent works involving the use of deep learning techniques and graph signal processing, one can mention the medical sciences [17]. Applications of GSP with ML for health have shown growth and been documented in a large number of works published in the literature [18]. In this scope, one identifies papers devoted to applications related to various medical specialties. There is evidence that some of these specialties, such as neurology, for example, have stood out in this context, while other areas are still little explored. The interest of researchers in using GSP and DL in neurology is due to the fact that the human brain can be modeled as a graph, so that its regions can be considered as vertices or nodes and its connectomes at functional and structural levels can be viewed as edges [19,20,21]. Deep networks, on the other hand, are widely used for automatic pattern recognition. In this context, the literature includes works dealing with different objectives, from early diagnosis of Alzheimer’s disease [22] and autism [23] to emotion recognition [24] and imagined speech [25] and multiple sciences [26].

In general, scholars in health sciences have demonstrated interest in the development and application of techniques simultaneously based on machine learning, signal processing, and graph theory. The interpretation and analysis of complex irregular data have potential to provide a number of benefits in clinical and hospital practice as an aid in identifying the origin of diseases, the early diagnosis of medical conditions, the verification of possible treatments, and disease prevention [27]. The elements outlined above encouraged us to prepare the present paper, which corresponds to a systematic literature review focusing on machine learning-based healthcare applications, with an emphasis on deep learning applied to signal processing over graphs. The paper presents an overview of the area: the medical specialties with the most papers in GSP in recent years, the ML and GSP techniques that have been most used in healthcare, the most influential authors in the area, and challenges, gaps, and open questions that may provide opportunities for future research. To be more specific, our paper includes the following:

- A comprehensive overview of ML and GSP applied to healthcare;
- A panorama of the datasets most used in ML applied to GSP in healthcare and their corresponding description;
- The identification of gaps, open problems, and promising future research directions in ML applied to GSP in healthcare.

**Figure 1:**
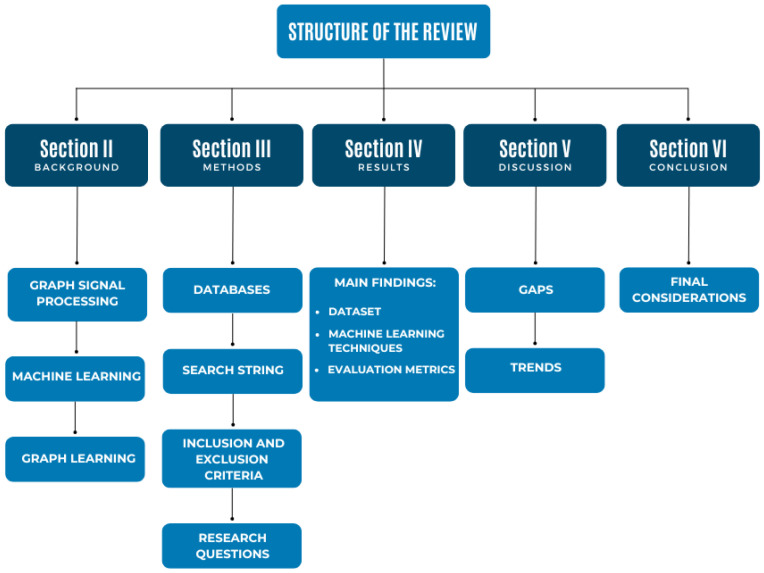
Organizational diagram of the paper.

The remainder of the paper is organized as follows. In Section 2, the basic fundamentals of graph signal processing, machine learning, and deep learning are presented. Section 3 corresponds to the methodology adopted for the systematic review, such as the scientific databases considered, the search strings used, as well as the inclusion and exclusion criteria of the papers. Section 4 presents the main findings of the review. Section 5 brings a discussion, in which the identified gaps are addressed and future research directions in the area are presented. In Section 6, the final considerations are presented. Figure 1 summarizes the organization of the paper.

## 2. Background

In this section, we provide a concise review of the main concepts related to graph signal processing and machine learning. In the case of GSP, the purpose is to explain what it means to consider a signal in the so-called vertex domain, as well as to indicate the main operators and approaches used in this framework. Regarding machine learning, besides listing the tasks that can be performed with its help and discussing some correlated issues, we highlight aspects of deep learning and the intersections of these tools with graph-based models.

### 2.1. Graph Signal Processing

Graph signal processing aims to extend classical digital signal-processing methods to signals over irregular domains represented by an arbitrary graph [27,28,29,30].

A graph is essentially a set of vertices (nodes) possibly connected by edges. Thus, each sample of a graph signal is associated with a vertex in the corresponding underlying graph; the edge weights reflect the interdependence among the signal samples [30]. In this context, the topology of a graph is inferred or determined according to the proposed application.

In terms of orientation, graphs can be directed, if the orientation of the input and output of the edge is considered, or undirected, in the opposite case. Another important characterization concerns the vertex degree. In the case of directed graphs, the vertex degree corresponds to the difference between the weight of edges that depart from it and the weight of edges that arrive at it. The degree of a vertex of an undirected graph, on the other hand, is the sum of the weights of the edges [31,32].

Additionally, a graph can be associated with an adjacency matrix, which is denoted by *A* and contains information about the connectivity of the corresponding graph. If there is an edge connecting vertices $v_{j}$ and $v_{i}$, the entry $A_{i,j}$ in the *i*-th row and the *j*-column of the referenced matrix is filled with the value of the respective weight; otherwise, $A_{i,j}=0$. An adjacency matrix is symmetric if and only if the associated graph is undirected. A graph can also be associated with a degree matrix, which is a diagonal matrix denoted by *D* and having in the entry $D_{i,i}$ the degree of the vertex $v_{i}$. Finally, the Laplacian matrix, denoted by *L*, is obtained by L=D−A [29,32].

In the study of graph signal processing, there are two well-established approaches [2]:

- Spectral graph theory: This is based on the graph Laplacian matrix and considers signals over undirected graphs with real and non-negative weights [1];
- Algebraic signal processing theory: This considers the adjacency matrix, which assumes the role of the elementary operator. This approach is used in signal analysis of directed and undirected graphs, which may have real or complex weights [30].

### 2.2. Machine Learning and Deep Learning

Machine learning corresponds to a subarea of artificial intelligence (AI), which is the field of study of systems that learn problems with examples obtained by training data [33]. Thus, ML aims to propose algorithms that can learn iteratively with the available data, in order to apply such algorithms to automate the construction of models capable of performing classification, regression, and clustering. These tasks can be based, for instance, on decision trees or artificial neural networks [34,35]. The use of such techniques has shown good results for applications in the most diverse areas, including medical diagnosis in health sciences [36,37,38].

In ML, two main approaches can be considered: supervised learning, in which training is performed considering labeled data, and the results of the model along the training are compared with the expected (target) outputs; unsupervised learning, in which the model identifies patterns in the data, with typical applications in clustering; in the latter, the data are not labeled and, as a consequence, there is no comparison between the output of the model and the target output along training [39].

Deep learning corresponds to a subarea of ML that makes use of deep neural networks. Such networks have high computational complexity and are widely used and disseminated for automatic pattern recognition [40,41,42,43,44,45]. DL techniques have been employed as an effective solution to perform pattern recognition in images, for instance. The most used approach, in this case, employs the so-called CNNs [46]. CNNs operate similarly to the receptive fields of the visual cortex of living beings and are essentially composed of convolutional, pooling, and dense layers [47]. A characteristic of this type of network is its high connectivity, which allows it to process a large amount of input parameters, as required in image processing [48,49].

However, CNNs are designed for data with a Euclidean structure. Nevertheless, as previously mentioned, there is a latent need to extend these techniques to the non-Euclidean domain, which can be accomplished by means of their generalization to graphs [50]. This gives rise to graph learning, a field of study that encompasses graph neural networks [51]. Moreover, considering the GL scenario, one has a specific GNN approach, the graph convolutional networks. Analogous to CNNs, GCNs have high connectivity to allow the input of a high number of parameters; in this case, the inputs are graphs [15,52].

## 3. Methods

The review presented in this paper encompasses papers written in English and published up to 30 October 2023. No starting date was defined for the search of papers in the literature. Four databases of relevance in the field of engineering were used: Science Direct, Institute of Electrical and Electronic Engineers (IEEE Xplore), the Association for Computing Machinery (ACM), and Multidisciplinary Digital Publishing Institute (MDPI).

The strings used for the search were as follows:

1. “Graph signal processing” AND (COVID OR disease);
2. “Graph signal processing” AND (health OR medical OR medicine) AND (“Neural Network” OR “Machine Learning” OR “Deep Learning”).

**Figure 2:**
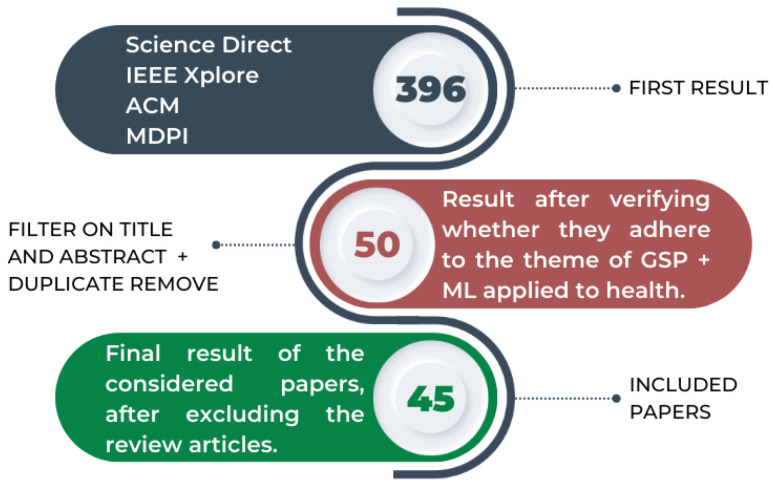
Flowchart of the paper selection process for the review considering exclusion criteria.

As a result, 396 papers were obtained. Refinements were performed to filter only the relevant papers for the purpose of this review. The first adopted strategy consisted of evaluating the title and the abstract of the papers and discarding those that did not adhere to GSP techniques applied to health. Additionally, repeated papers were also subtracted, so that 50 papers remained for analysis. Finally, 5 more papers were disregarded because they were review papers. As shown in Figure 2, a final sample of 45 papers remained for analysis.

It is worth mentioning that five review papers were found, which substantially differ from our paper, both in scope and in selected works, and consequently in their findings.

In Khambhati et al. [53], for example, the selected papers concern specifically graphs on dynamic patterns of brain connectivity. In the paper of Dong, Wang, and Abbas [54], the review addresses works in the literature that use deep learning. It is not a review on graph signal processing, although there is a section dedicated to the subject. The paper by Li et al. [55] is a review on graph signal processing and neural networks in the biological data scenario. In this case, despite being a broader review, it is a study more aligned with the biological sciences, since it includes the study of molecules and proteins.

The paper by Yin and Kaiser [56] addresses neural flexibility in the human brain. To this end, they reviewed the computational approaches and suggested metrics to classify the flexibility of brain regions. In the work by Yingjie et al. [57], a specific area of the health sciences is analyzed: the work is concerned with the use of deep learning to diagnose liver diseases, and among the methods considered, one observes graph neural networks to detect liver tumors.

Unlike the aforementioned papers, our work is in the field of health in general, without a restricted medical area or specialty; we address papers on methods that use machine learning for graph signal processing in health.

After the paper selection and exclusion stages, the most relevant characteristics for carrying out the analysis of the 45 selected papers were extracted and synthesized. The information considered in the analysis are those related to the nature and metadata of the paper:

- Year of publication;
- First author’s country of affiliation;
- Studied area.

Other issues considered in the analysis were the following:

- Dataset (size, type, and characteristics of sample);
- Proposed technique versus the technique used for the comparison;
- Objective of the study;
- Performance metrics.

The works were analyzed, and gaps and open challenges were identified. The results of such an analysis can serve as guidelines for future work in the area.

## 4. Results

**Figure 3:**
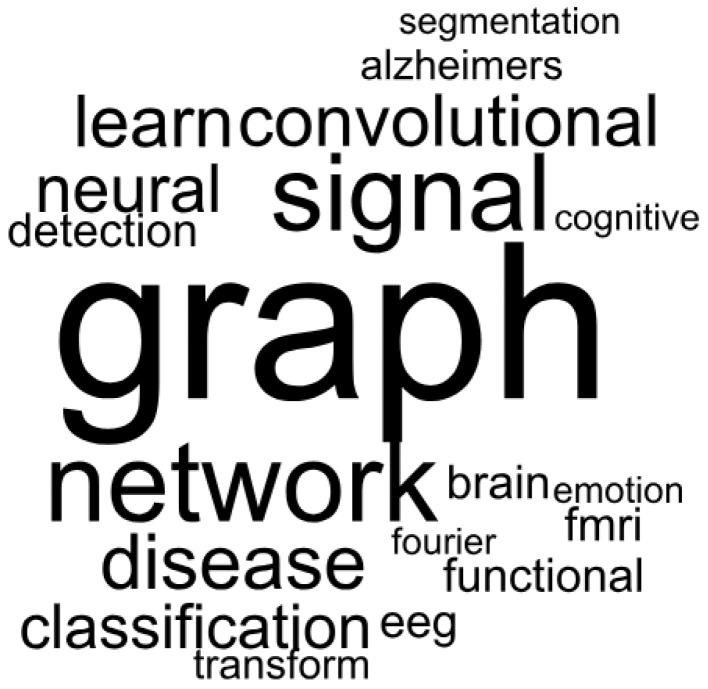
Word cloud obtained from the titles of the 45 papers using the Iramuteq software.

Initially, lexical analyses were performed on the 45 papers included in this review. The analyses were based on the frequency of occurrence of terms in the titles and the keywords. One of these analyses is the word cloud, which consists of a simplistic visual representation to highlight the words with high recurrence in a previously defined universe [58,59]. Then, the larger the size of the word in the cloud, the more times it occurs in the text. An analysis of this type is depicted in Figure 3, which was obtained to show the co-occurrence of terms in the titles of the papers. In the presented analysis, the terms with the largest font are the most frequent ones in the area under investigation. This study was carried out using the Iramuteq software [60], which is free to use and was developed as open source using the R and Python languages. It can be inferred that terms related to GSP and DL appear very often, as expected, but there is also a considerable occurrence of terms related to neurology, such as: “fmri”, “eeg”, “brain”, and “alzheimer”.

One of the encountered issues in the use of word clouds is the lack of grouping of similar terms because of grammatical variations, such as singular and plural [61]. In Iramuteq, this question is solved by the use of textual lemmatization. Thus, a certain level of variation is allowed in the terms, so that they are not considered distinct and the occurrence count is added to its most frequent equivalent term [62,63].

Another analysis that can be carried out with the Iramuteq software is the similitude analysis [64], which is based on graph theory. In this case, the most important words in the analysis are represented by vertices of a graph structure and the connections between words correspond to the edges. Thus, it is possible to identify central terms, their connections, and the grouping of words of the same theme just like a hypergraph.

**Figure 4:**
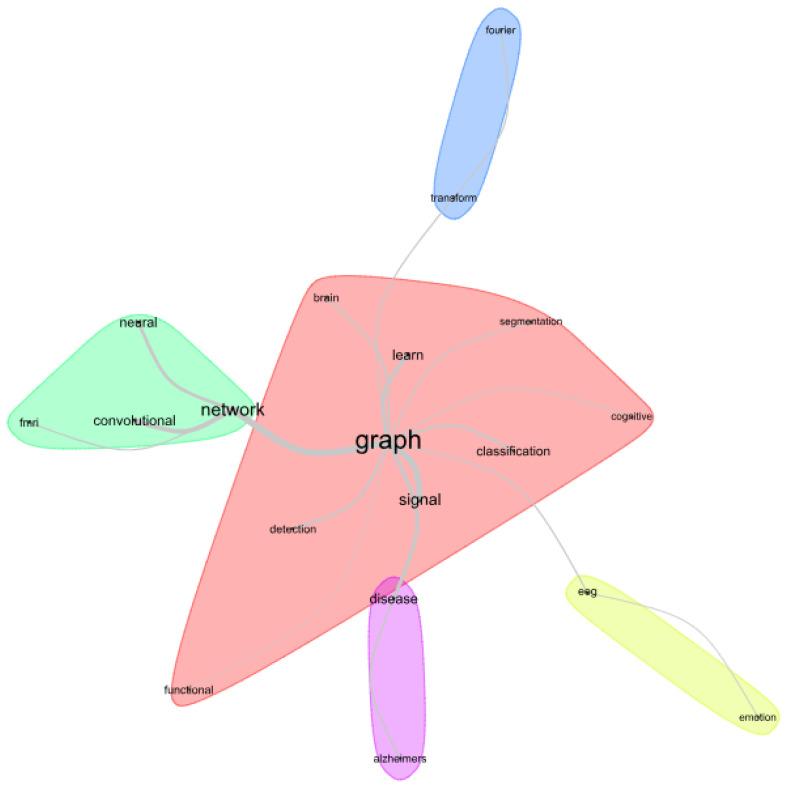
Similitude analysis obtained from the titles of the 45 papers using the Iramuteq software (0.7 alpha 2).

Figure 4 shows a similitude analysis obtained by Iramuteq for the titles of the papers. The figure shows a central cluster with words that are frequently related; such a main cluster is connected to other clusters through its secondary terms. As a central term, one observes the word “graph”, as expected. From this, branches are shown with clusters of distinct themes, but originated from the central elements.

**Figure 5:**
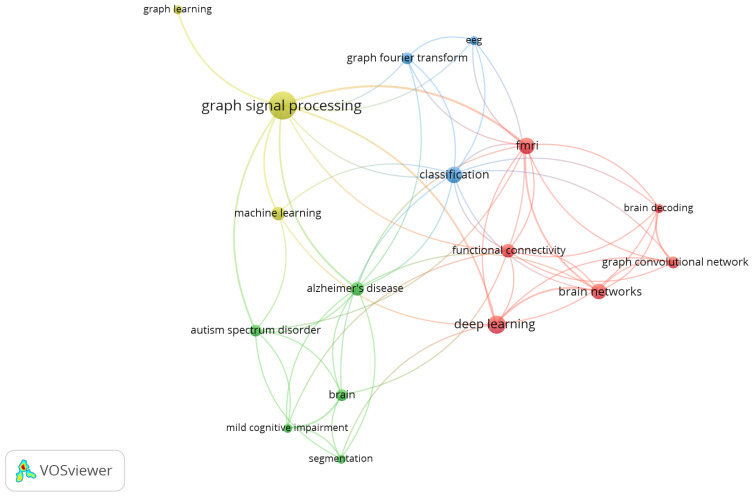
Graph analysis considering the occurrence of keywords.

Figure 5 allows a complementary analysis. In this case, the co-occurrence of keywords is evaluated with the VOSviewer software, which is a tool for the elaboration of bibliometric networks [65]. The most recurrent terms are “graph signal processing”, “deep learning”, “graph learning”, “machine learning”, “fmri”, “connectivity”, “Alzheimer’s disease”, “autism spectrum disorder”, “brain”, “mild cognitive impairment”, and “graph fourier transform”. The nodes were divided into four clusters, so the most frequently related terms are grouped together in the same color. According to the terms shown in the figure, once again as expected, the application focused on neurology is highlighted in the terms in evidence.

**Figure 6:**
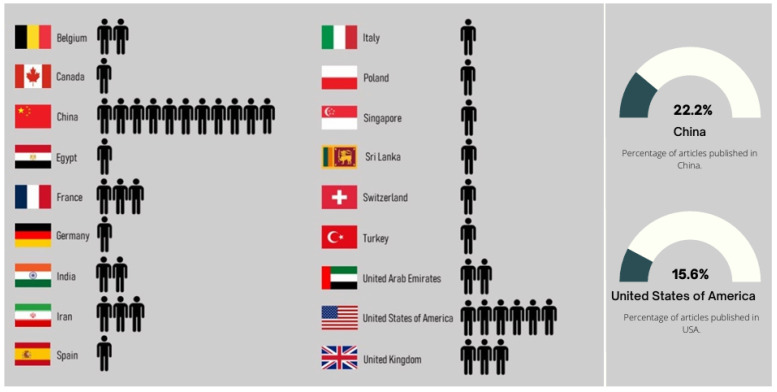
Country associated with the affiliation of the first author of the papers included in the review.

**Figure 7:**
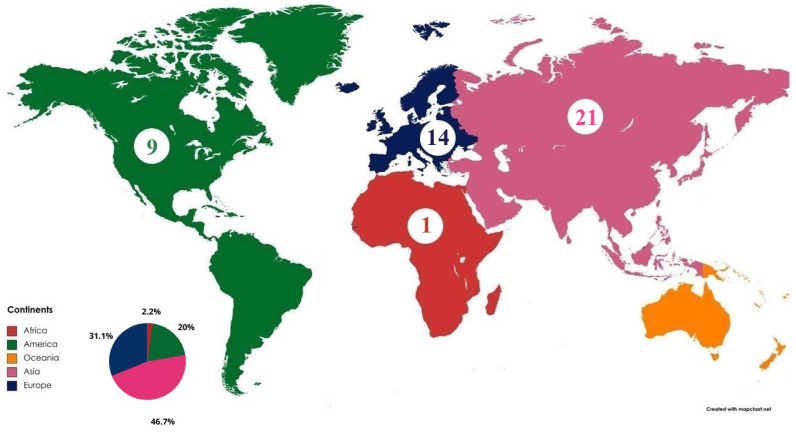
Continents associated with first author affiliation of the papers included in the review. The continents are separated by color and the numbers indicate the number of publications per continent.

The distribution of publications by geographic location took into account the country associated with the affiliation of the first author of each paper. This made it possible to analyze the paper distribution by country and by continent, as shown in Figure 6 and Figure 7, respectively. In Figure 6, we verify that there are first authors affiliated with institutions from seventeen different countries, with emphasis on China, the United States of America (USA), Iran, and the United Kingdom; the first two countries have, respectively, ten and seven, and the last two countries have four, of the forty-five first authors.

Figure 7 presents the geographical overview from a continental point of view. It can be inferred that there is at least one first author per continent, except in Oceania. The continent with the greatest influence is Asia, corroborating the strong impact provided by China. It is followed by the European continent, which has the United Kingdom and France among the most influential countries according to the number of affiliated first authors. The next continent in this sequence is America, which, despite the strong influence of the USA, has only one other country with two affiliated first authors, Canada. Among the continents with publications, the last is Africa, with only one first author. Europe and Asia together hold 77.8% of first author affiliations.

**Figure 8:**
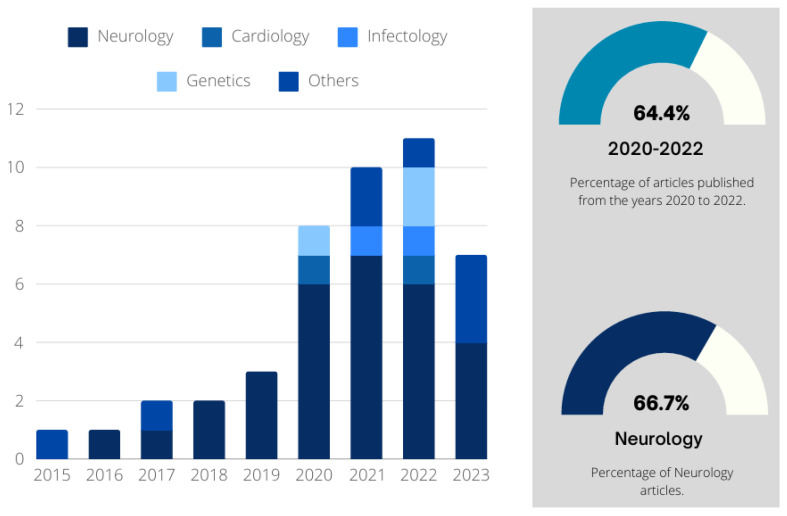
Distribution of publications by year and medical specialty.

The trend of publications by year was also analyzed in this paper. As illustrated in Figure 8, among the 45 considered papers, the first one was published by Toutain et al. [66], in 2015, being the only paper that year. In 2016, there was again only one publication. In 2017 and 2018, the number increased to two publications per year. In 2020, with eight papers published, the growth was 166.67% compared to the previous year. A growth in the number of publications was observed in 2021, when ten papers were published. In 2022, one observes eleven publications. It can also be inferred that the recent development of the research field that makes use of GSP and ML techniques is evident, which can be observed with the beginning of publications in 2015 and the growth in subsequent years.

Figure 8 also presents the number of papers published per year by specialty; it corroborates the emergence of papers that use GSP, ML, and DL for neurology applications, which represents 66.7% of the 45 evaluated studies. However, it is evident that the research field that makes use of GSP and DL techniques is very recent, since the first paper found in this study was published in 2015. On the other hand, it can be said that the area is under consolidation, with the remarkable growth in the number of publications in recent years: in the period from 2020 to 2022, 64.4% of papers were published.

**Figure 9:**
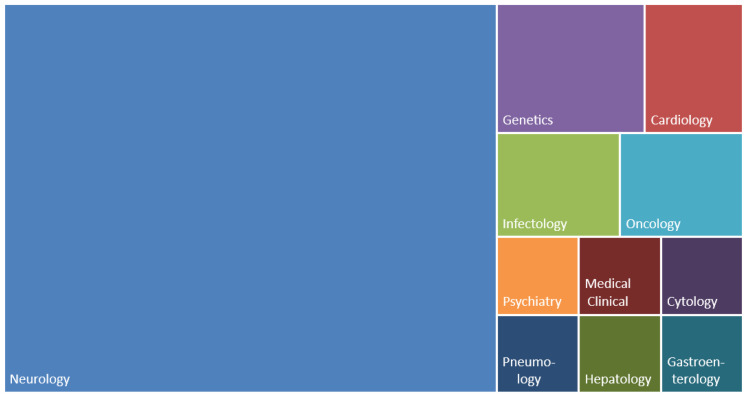
Tree map with the distribution of papers by medical specialty.

Figure 9 shows the eleven areas with publications by means of the tree map, in which the sizes of the squares of the specialties are proportional to the number of publications. Thus, considering the universe of 45 papers selected for this review, neurology is the most prominent (30), followed by genetics (3), cardiology (2), infectology (2), oncology (2), gastroenterology (1), medical clinic (1), cytology (1), psychiatry (1), pneumology (1), and hepatology (1).

**Figure 10:**
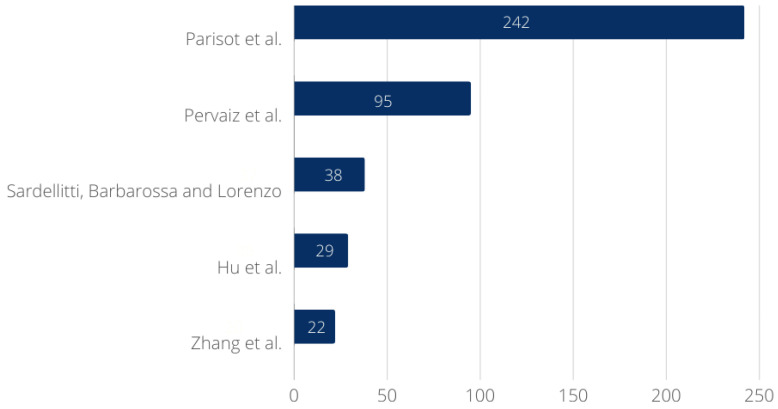
The five most cited papers according to the Web of Science database.

Figure 10 shows a bar chart of the number of Web of Science citations of the five most cited papers. The paper by Parisot et al. [67] is indicated as the most cited, with 242 citations. Pervaiz et al. [68] ranks second, with 95 citations. There are 38 works that use the study by Sardellitti, Barbarossa, and Lorenzo [69] as a reference, a number reasonably close to the fourth most cited, the work by Hu et al. [70], which has 29 citations. Finally, Zhang et al. [71] ranks fifth, with 22 citations. It can be inferred that there is a considerable difference in the number of citations of [67] compared to the others, which may indicate this work as recommended reading in the area.

**Figure 11:**
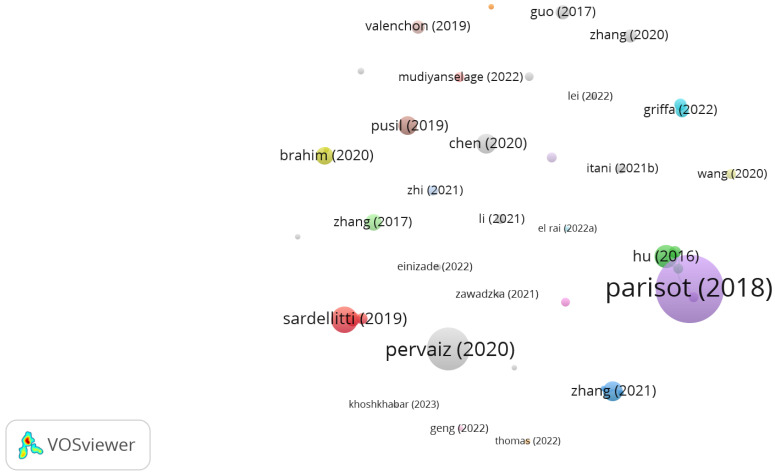
Most cited papers represented by the first author.

Figure 11 shows a map of citations obtained with VOSviewer [72]. The map is made up of spheres, labeled with the names of the first authors of the most cited papers and with sizes related to the number of citations received. It is also possible to see the five most cited papers, as shown in the previous figure. In general, the other nodes have similar sizes, indicating that they have received a similar number of citations, reaching a maximum of 21.

**Figure 12:**
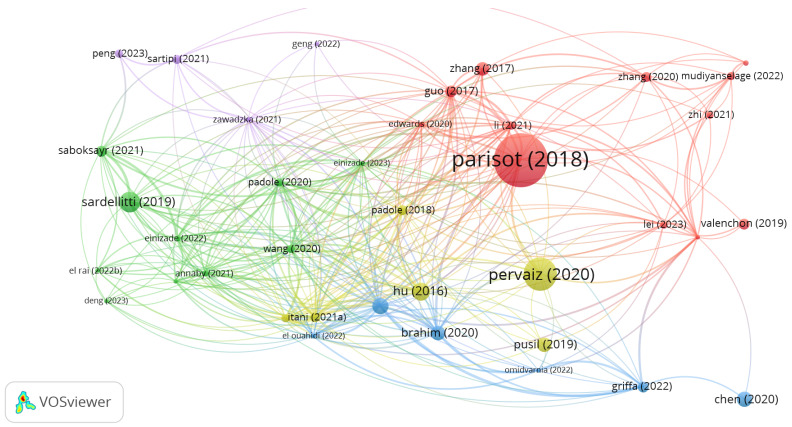
Graph network representation for bibliometric coupling analysis.

Figure 12 shows a bibliometric coupling network obtained through analysis in VOSviewer. The nodes of the graphs represent the first authors of the papers, and the size of the vertex is related to the number of citations of the paper. The edges connect the nodes that are bibliographically linked when there is another publication that is cited by the simultaneously linked papers.

In order to establish a relationship between each paper and its respective application in the studied health area, Table 1 allocates the 45 evaluated papers to their respective specialty among the eight identified specialties.

Table 2 presents the main information extracted from the studied papers. The presented descriptive data refer to the year of publication, the objective of the developed research, the technique proposed in the paper, and with whom it was compared to in order to evaluate its performance. Table 3 presents a set of information on the dataset used in the selected studies, such as the dataset used, the sample size used, and finally, the metric used to evaluate performance.

**Figure 13:**
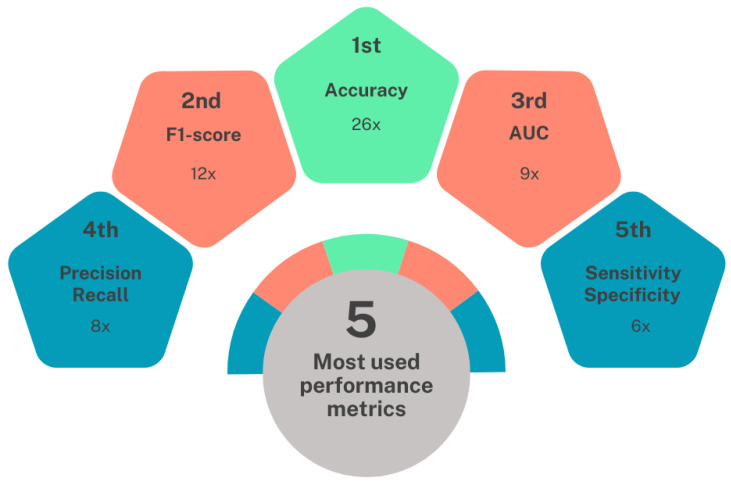
Diagram with the 5 most frequent performance measures in the works analyzed.

According to Table 3, it is possible to identify the five most used metrics in evaluating the performance of the proposed models, as shown in Figure 13. Accuracy occupies the first place. It is used to assess performance in 26 out of the 45 studies analyzed. In the second place, we observe the F1-score, which appears in 12 works. The AUC holds the third position. It is used in nine papers. In the fourth position, the measures precision and recall are tied. They are used in 8 out of the 45 selected papers. Finally, in the fifth position, we have sensitivity and specificity, which were used in six works.

**Figure 14:**
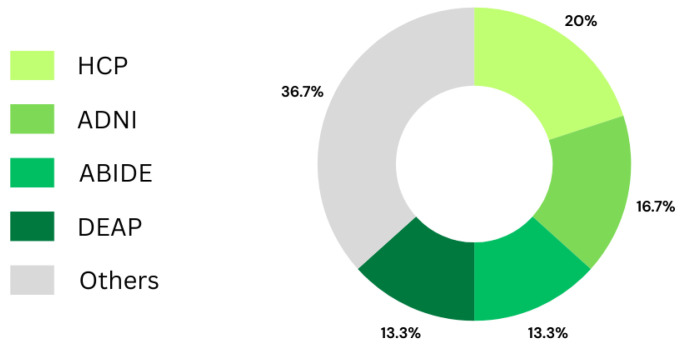
Graph showing the most frequently used databases in neurology.

Another analysis obtained from Table 3 concerns the most used databases. Considering the area of neurology, which corresponds to 30 of the 45 articles included in the review, Figure 14 shows that the most used databases were HCP, used 6 times, followed by ADNI with 5 uses, and then ABIDE and DEAP, which were used in 4 papers each. It is important to note that those databases are publicly available.

## 5. Discussion

One of the challenges reported by the analyzed papers is related to the difficulty of accessing health-related datasets. The limited amount of data (whether images, signals, or medical records, among others) may lead to a lack of generalization of the proposed approaches in the detection or classification of pathologies. Another challenge is related to the reproducibility of research, since different research groups are unable to evaluate new methodological proposals for the reported problem if a common dataset is not available. There is need for more publicly available datasets.

Among other limitations addressed by the papers, we can mention data imbalance. In [67], for example, it is mentioned that, in future studies, one of the intentions is to verify the use of graph convolutions to achieve good prediction rates in problems that present data imbalance, since it is considered a factor that hinders the learning of intelligent systems.

Regarding the dataset, one possibility to achieve better performances would be to include complementary information to signals or images. This is due to the fact that, in health-related problems, it is relevant to use dataset with a combination of data, such as phenotypic information, because diagnoses may be related to morphological characteristics or conditions and clinical parameters. In [78], for example, it is stated that the work has a limitation because it uses only brain image data; better results could be obtained if the referred additional information would have been employed.

Another issue to be considered concerns the medical interpretation of the results obtained by systems using GSP and computational intelligence techniques. Although many proposals achieve good performance, considering the evaluation of objective metrics, which are quite widespread in engineering, it is of paramount importance that there is an understanding of the addressed problem, based on the understanding of what the result means, and also how it impacts the analysis in the health sciences, in order to achieve a broader and more complete analysis for diagnosis. In this context, Valenchon and Coates [80] report their intention that, in further research, rather than the outcome of the proposal indicating whether or not an individual with dementia will progress to Alzheimer’s disease, it presents a mechanism that provides a value of the probability of progression, which guarantees a more complete medical analysis. Another example is [68] in which it is suggested that future work may address possibilities beyond the prediction of clinical diagnosis, one of them being to investigate and suggest possible treatments for the found medical condition.

In relation to this, an area that addresses such issues and presents possible solutions to minimize these difficulties is explainable artificial intelligence (XAI) [145]. This is a recent field that concerns the explanations and interpretability necessary in processes that use artificial intelligence techniques in predictions, so that there are justifications for and credibility of the obtained results [146]. In any case, interpretability and explainability are terms that encompass standards and criteria, which must be taken into account according to the associated context [147]. In the case of this review, the context to consider is the medical specialty of the application, and then make the proposal based on computational intelligence understandable to health professionals, knowledgeable about the nature of the problem, reducing the gap between the proposal and clinical practice. In [79], for example, a system for detecting and evaluating signals of neurological examinations for the early detection of Alzheimer’s disease was developed; according to the authors, it would be interesting for health professionals to understand the approach devoted to signal detection, with the aim of enabling the use of programs that validate the proposal in a real medical context.

In this context, such issues fall under health 4.0 (H4.0), a term used to relate health advances to industrial technological revolutions. It corresponds to a field that investigates the use of technologies in favor of patient care, based on the use of technology to promote better and faster diagnostic capacity, equipment portability, and greater data management capacity [148]. Thus, the use of technology is aimed at clinical care itself, and can be supported with the use of artificial intelligence, including deep learning techniques for care aimed at early diagnosis, the prevention of the progression of health conditions, and early identification of effective treatments [149,150].

An important challenge verified in the analysis of the papers included in the review is the extraction of characteristics from the data used, since this step is not restricted to the extraction itself, but to the selection of more relevant characteristics so that the proposed system for the intended application is able to identify health changes due to the selection of more significant characteristics. In this case, the use of convolutional networks can be considered, since the convolution layers play the role of the extractor of features.

Another challenge is the selection of optimal hyperparameters for the proposed techniques, because although they present a good performance, as reported in [22], it is possible to achieve superior results with an assertive selection of hyperparameters. The choice of hyperparameters can be made using grid search, Bayesian optimization, or random search and swarm intelligence.

Many applications addressed in the selected papers are in neurology, in which the data evaluated are examinations converted into time series. In this sense, an important analysis to aid diagnosis is to check the regularity of the series and identify noise. That analysis can be carried out by using information theory metrics on graphs, such as permutation entropy and dispersion entropy [151,152]. This could be a promising research area in graph signal processing.

Regarding the works in which the specialty of neurology is considered, a frequently considered analysis employs functional connectivity. Therefore, accurate pattern recognition is essential, which can be achieved through the use of robust graph learning techniques, acting in the identification and analysis of connectivity between brain areas. Another possibility of analysis is structural connectivity. According to [75], in future work, a specific structural connectivity for each individual should be considered. This would lead to the definition of multiple spectral domains for brain signals, and would enable the analysis of inter-subject structural variability. Still in this specialty, many studies report the use of atlases to divide the brain into areas. However, there is no consensus on the use of a single atlas to carry out the referred division. It would, therefore, be interesting to verify and test the use of different atlas options for the same dataset, since this choice has a high potential impact on the final classification stage.

Due to the good results presented with different techniques that combine GSP and ML, it is possible to use graph neural networks and test the proposed methods in different medical applications of high complexity and that have data available in the literature, as suggested in [66,79]. Considering high-complexity problems, one of the future proposals reported in [95] concerns real-time processing for echocardiogram videos. This could be a major advance in early diagnosis with artificial intelligence, and would represent a significant impact for health sciences.

The proposal described in [74], which employs the modified Laplacian matrix to classify attention deficit hyperactivity disorder, presents a promising result, so its use can be considered as an alternative mathematical framework in other medical applications. Likewise, in [103], it is recommended to explore the potential of Multi-GNNs, which consist of combining the characteristics of individual GNNs.

An interesting consideration concerns the use of new transforms, because, although the Fourier transform is quite widespread and leads to good results, it is important that different transformation techniques be examined and tested. In [88], for example, the investigation of new transforms is pointed out as a future proposal, as the authors mention the fact that new transformation techniques can lead to improvements in the classification rates.

Finally, an important issue is the lack of standardization, so it would be interesting to standardize metrics and evaluation techniques for comparison purposes.

## 6. Conclusions

In healthcare, GSP has been used to analyze problems related to signals lying in non-Euclidean domains. In addition, ML techniques have been used for pattern recognition and early disease classification and identification. Considering the 45 papers included in the systematic review, 30 of these presented applications for neurology problems, with many of them focused on the diagnosis of cognitive impairment and Alzheimer’s disease. In these cases, most of the data correspond to fMRI and EEG images. However, limitations are reported regarding the number of samples and the number of publicly available dataset.

From the presented data regarding the number of publications, it is clear that, despite GSP with ML applied to health being a recent field of study, it has shown an increase in the number of publications, which may indicate an interest of the scientific community in the area. Advances in the scope of GSP with ML in health have attracted the attention of health professionals, since the proposed methods have a high capacity to assist early diagnosis and, consequently, provide speed in decision making by specialists. In any case, there are gaps to be solved, such as a better integration between computational intelligence techniques and clinical practice.

This systematic review synthesized the information from selected papers and pointed out the trends of applications that are emerging in the area, as well as methodologies that combine artificial intelligence, graph theory, and health sciences, presenting subsidies for researchers to explore gaps in future work, as well as to reproduce existing work. A limitation of this work is the number of scientific databases considered. Although our study has considered four relevant scientific databases (IEEE Xplore, Science Direct, MDPI, and ACM), it is possible that there are other papers that fit the scope of the review and that have not been included. It is also possible that new papers have been published after the period defined for the inclusion of papers, which was October 2023.

In the future, further updates of this literature review can be carried out, including more databases and also revisiting those considered in this paper, since, with the identified trend of publications, there should soon be new research published in the area.

## Figures and Tables

**Table 1:** Distribution of the papers according to the medical specialty related to the proposed application.

Medical Specialty	Ref.
Neurology	[22,23,24,25,67,68,69,71,73,74,75,76,77,78,79,80,81,82,83,84,85,86,87,88,89,90,91,92,93,94]
Cardiology	[95,96]
Infectology	[97,98]
Genetics	[99,100,101]
Oncology	[102,103]
Gastroenterology	[104]
Medical Clinical	[105]
Cytology	[66]
Psychiatry	[106]
Pneumology	[107]
Hepatology	[108]

**Table 2:** Information from the papers included in the systematic review.

Ref.	Year	Objective of the Study	Proposed Technique	Techniques Used for Comparison
[66]	2015	Image preprocessing, segmentation, and classification of Feulgen- and Papanicolaou- stained slides.	Partial difference equations on weighted graphs.	Graph cuts, random walks, shortest-path algorithms, maximum spanning forests, and power watershed algorithm.
[79]	2016	Alzheimer’s detection.	Matched signal detection (MSD) theory for signals on graphs (simple-MSD, constrained-MSD, probabilistic-MSD).	Principal component analysis (PCA), support vector machine (SVM), and linear discriminant analysis (LDA).
[82]	2017	Brain image data modeling and extraction.	Autoencoders for analysis of high-dimensional graph signals.	PCA, robust PCA (RPCA), graph-based filtering (GBF), and stacked autoencoder (SAE).
[105]	2017	Prediction of risk of comorbidities.	Heterogeneous convolutional neural network (HCNN), based on predictive learning.	Logistic regression (LR) and standard CNN.
[67]	2018	Autism and Alzheimer’s classification	GCN for population analysis.	Random forest (RF) and multi-layer perceptron (MLP).
[78]	2018	Alzheimer’s detection.	Graph frequency analysis for highly discriminative feature extraction and GCNN-based classifier.	MSD-G [79], RsBN-DL [109], Sparse-Cov [110], and EN-LogReg [111].
[69]	2019	Orthonormal data transformation applied to images of patients with epilepsy.	Orthonormal sparsifying transform and graph Fourier transform (GFT).	SpecTemp [112], Kalofilias [113], and Dong et al. [114]
[80]	2019	Alzheimer’s detection.	Multiple feature-specific adjacency matrices for learning using GCNN.	Linear SVM, MLP, RF, Parisot et al. [115], and Vivar et al. [116].
[89]	2019	Predicting cognitive impairment in Alzheimer’s disease (AD).	Multifrequency dynamic network analysis for building a connectome biomarker.	PCA.
[83]	2020	Attention deficit hyperactivity disorder (ADHD) detection.	GSP and GL to obtain structural and functional characteristics.	MLP with double input symmetrical relevance (DISR) and MLP with minimum redundancy maximum relevance (mRMR).
[77]	2020	Detection of central brain regions.	GFT based on Laplacian learning for analyzing graphs in the frequency domain.	Radial basis function (RBF) kernel and Pearson correlation methods for calculating the graph Laplacian.
[22]	2020	Alzheimer’s detection.	Graph coarsening in a GCNN.	Heavy Edge [117], Kron Reduction [118], and spectral approximation [119].
[74]	2020	ADHD classification.	Dual-subspace classification algorithm using individual resting state functional connectivity.	RMf, fusion fMRI, R-Relielf, L1BioSVM, FCNet, 3D-CNN, and Deep fMRI
[73]	2020	Autism classification.	GFT and ML for analyzing the test and time series to calculate descriptive statistics for the region of interest.	[120,121,122,123,124,125,126].
[68]	2020	Classification of neurological function.	Graph-based modeling of the brain’s functional connectivity with elastic net and independent component analysis (ICA).	RF, Dictionary Learning, and Higher Dimensional YEO parcellation.
[100]	2020	Prediction of RNA association with disease.	Graph attention adversarial network (GAAN), based on the integration of state-of-the-art GCN and the attention mechanism.	Ding’s method [127], RWRMDA [128], TPGLDA [129], RLSMDA [130], GCN, GAT, and GAN.
[96]	2020	Aortic root segmentation.	Multi-resolution graph using irregularly spaced patch sampling and a graph-based CNN as a classifier.	Hand-crafted and Fully connected graph.
[102]	2021	Gene selection for cancer detection.	Algorithm for selecting significant genes with GSP techniques, using the Laplacian matrix of the graph.	Locally linear embedding (LLE) and PCA.
[81]	2021	Emotion recognition.	Spatio-temporal attention neural network with GFT signals as input.	Multi-column convolutional neural network (MCNN) [131] and bidirectional long short-term memory (BiLSTM) [132].
[23], [84]	2021	Autism classification.	Connectivity matrix with GFT values, extension of the Fukunaga–Koontz transform for feature extraction to train the decision tree (DT).	Spatial filtering method and the GFT.
[103]	2021	Lung cancer detection.	Multi-graph neural network (MGNN) with three models: GIAN, GIAT, and SGCA.	ML algorithms, RF and support vector regression (SVR).
[71]	2021	Multidomain brain decoding.	Multidomain decoding model on short time series incorporating Laplacian graph with GCN.	Classical brain decoding model, which applies multi-class linear SVM.
[97]	2021	Identification of the focus of disease spread.	GSP, GCN, and neighborhood loss calculation to optimize the average error distance.	Label propagation framework for source identification, Unbiased Betweenness.
[88]	2021	Motor imagery classification.	Graph-theoretic models of multichannel EEG signals with multivariate autoregressive models for directed graphs and extreme learning machine classifiers.	SVM, K-nearest neighbor classifiers (KNN), and Extreme Learning Machines (ELMs).
[90]	2021	Emotion classification/epileptic seizure analysis.	GFT for the extraction of discriminative features used in learning tasks and the proximal gradient method for data acquired in real time.	[113,133] and SVM.
[91]	2021	Emotion recognition and analysis.	GSP to integrate emotion recognition and analysis of signals.	–
[25]	2022	Brainwave decoding.	Fusion of GSP and GL resources for a method called graph-based imagined speech BCI decoder (GraphIS).	–
[75]	2022	Task decoding and individual fingerprinting	SVM classification and GSP functional data filtering for functional connectivity and structural connectome decomposition.	–
[85]	2022	Elimination of noise from epileptic EEG signals.	Unified objective function for GraphJADE with GL and use block coordinate descent to optimize it.	Unified objective function GraDe with GL and the blind separation methods.
[95]	2022	Left ventricular segmentation in echocardiography videos.	GraphECV with GSP for semi-supervised learning and minimization of the Sobolev norm of graph signals.	PReMVOS [134], TMANet [135], Accel [136], and OSVOS[137].
[86]	2022	Evaluation of how brain activity changes over time.	GSP, SVM, and multiscale entropy.	–
[87]	2022	Brain Activity Classification.	End-to-end GCN structure with three convolutional layers.	NetMF [138], RandNE [139], Node2Vec [138], and Walklets [140].
[99]	2022	Detection of metabolic diseases.	GCN to infer potential metabolite–disease association, named MDAGCN.	Traditional methods based on biological experiments.
[98]	2022	Study on the contagion dynamics of COVID-19.	Wavelet transform of spectral graph to process data in dynamic graph for spatio-temporal pattern detection.	–
[101]	2022	Prediction of circRNA association with diseases.	Two GCN-based prediction models: Node Classification and Link Prediction.	Other baseline models.
[104]	2022	Early diagnosis and detection of gastrointestinal polyps.	Semi-supervised segmentation called SemiSegPolyp, based on GSP. It is divided into instance segmentation, construction of graphs based on nearest neighbors, and semi-supervised semantic segmentation.	Mean-Teacher, generative adversarial networks (GANs), Cross-Consistency Training, and Wu [141].
[93]	2022	Understanding what are the most useful graph frequencies to decode fMRI signals.	Spectral ResNet, in which the frequencies of the graphs define the convolutions.	MLP pattern, where the input domain is the frequency domain of the graph.
[76]	2023	Detection of mild cognitive impairment (MCI).	Multiscale enhanced GCN.	SVM, two-layer GCNs, and multi-scale GCN with the same normalized adjacency matrix.
[92]	2023	Clinical follow-up to assist in the diagnosis of inflammatory bowel diseases.	GFT, GSP, and classical SVM are used to classify the features.	Graph theory analysis method.
[24]	2023	Emotion analysis in EEG.	Coding of relative temporal transformation and attention to the channel.	GCNN, SVM, CNN + recurrent neural networks (RNNs).
[94]	2023	Classification of sleep stages.	Adaptive GCN, named ProductGraphSleepNet, which exploits GSP and product graph learning (PGL).	SVM, RF, MLP+LSTM, DeepSleepNet, CNN, RF+ Hidden Markov Model (HMM), U-Sleep, SeqSleepNet, SleepECL, fractional Fourier transform (FRFT), catBoost, LR, second-order blind source separation (SOBI)-wavelet Transform (WT), ProductGraphSleepNet, SSL-ECG, SimCLR, TS-TCC, time–frequency features, multitaper spectral + CNN, intra-/inter-epoch BiLSTM, FRFT, NAS, Cascaded CNN+LSTM.
[106]	2023	Discover how default mode network (DMN) alignment is related to symptoms of depression and rumination.	Graph signal processing-based analyses in a transdiagnostic cohort.	-
[107]	2023	Evaluation of the quality of the photoplethysmography (PPG) signal.	Analysis of graph signals using six machine learning classifiers: RF, DT, SVM, MLP, CNN, and Naive Bayes (NB).	Comparison of the six classification techniques mentioned.
[108]	2023	Identification of liver organs and segmentation of liver tumors.	Simple Linear Iterative Clustering (SLIC) algorithm for clustering liver computed tomography (CT) images and convolutional graph networks with four Chebyshev graph convolution layers and one fully connected layer to detect liver organs and segment liver tumors.	Modified U-Net and Shortcut CNN.

**Table 3:** Summary of information regarding the dataset used and metrics considered in the performance evaluation of the proposed models.

Ref.	Dataset Used	Dataset Description	Evaluated Metrics
[66]	GrabCut, MNIST, OPTDIGITS, and PENDIGITS.	MNIST, OPTDIGITS, and PENDIGITS datasets are composed of handwritten digits.	Error measures and classification rates.
[79]	PIB-PET dataset and ADNI.	PIB-PET dataset is composed of PET neuroimages and consists of 30 patients with Alzheimer’s disease (AD) and 40 healthy control (HC) subjects; ADNI dataset is public and consists of resting-state fMRI, containing images from 30 individuals with early MCI and 20 NC subjects.	Accuracy, sensitivity, specificity, and area under the curve (AUC).
[82]	Real MEG datasets.	MEG signals collected by 306 sensors were considered. Brain activity was captured by the participants’ reaction to seeing 322 images of human faces and 197 images of objects that were shown randomly.	Accuracy.
[105]	Electronic Health Record (EHR) data.	The data consist of the medical records of 3048 patients with congestive heart failure; 18,451 with diabetes; 3948 with chronic kidney disease; 7700 patients with chronic obstructive pulmonary disease.	Precision, recall, and F1-score.
[67]	ABIDE; ADNI.	ABIDE is a public dataset of functional NMR and phenotypic data. It considered 403 individuals with spectrum disorder and 468 HC; in ADNI, 1675 samples were available, with 289 individuals (843 samples) diagnosed with AD.	AUC.
[78]	ADNI.	It considered 100 subjects with MCI and 100 HC subjects.	Accuracy.
[69]	One synthetic dataset and one real dataset [142].	The real dataset has only one epilepsy patient and 76 time series.	Correlation coefficient, percentage of recovery errors, F1-score, precision, and recall.
[80]	TADPOLE.	779 subjects, 296 MCI converters, and 483 MCI non-converters.	AUC.
[89]	Collected for the paper.	MEG recordings were obtained in 54 patients with MCI aged 65-80 years. They were divided into two groups according to their clinical outcome: (1) the “progressive” MCI group (N=27) was composed of the individuals who met the criteria for probable AD; (2) the “stable” MCI group (N=27) was composed of the participants who still met the criteria for a diagnosis of MCI.	Classification performance, sensitivity, and specificity.
[83]	Online dataset.	Public dataset with EEG signals from normal and ADHD children aged 7–12 years.	Accuracy.
[77]	Collected for the paper.	Task-based resting-state fMRI images. The participants were divided into two categories: young adults, aged 18–22 (119 women, 79 men); children, aged 8–12 (108 women, 83 men).	F1-score, recall, and precision.
[22]	ADNI.	Public, over 800 participants, including HC individuals with MCI and individuals with AD. The dataset included several classes of imaging: structural MRI, functional MRI, and PET scans, as well as clinical and cognitive assessments.	Operator dissimilarity index and cut index.
[74]	TDAH-200.	The resting state fMRI (rs-fMRI) data used to investigate the binary classification performance between ADHD and HC subjects.	Accuracy.
[73]	ABIDE.	fMRI images of 871 subjects were considered, 403 subjects with autism spectrum disorder (ASD) and 468 HC.	Accuracy, sensitivity, and specificity.
[68]	UKB; HCP.	fMRI data from the UK Biobank (UKB), which consists of 13,301 individuals; HCP of 1003 HC.	Accuracy/correlation.
[100]	HMDD; LncRNADisease.	HMDD is a public dataset on miRNA diseases. A miRNA–disease network with 208 miRNAs, 250 diseases, and 3644 links was considered; LncRNADisease dataset is public and provides information on lncRNAs and diseases with over 200,000 lncRNA–disease associations across 529 diseases and 19,166 lncRNAs.	AUC and prediction results.
[96]	An example on aortic valve.	Human torso CT samples are considered for studying the aortic root.	Accuracy.
[102]	Three datasets [143].	Public genetic datasets. In the prostate cancer dataset, there are 50 normal prostate samples and 52 prostate tumor samples, each sample with 10,509 different genes. The gastric cancer dataset contains 40 samples, 20 of which are from normal patients and another 20 from gastric cancer patients, each sample with 10,519 genes. In the brain dataset, two classes are considered, both brain tumors, glioblastoma with 20 samples and oligodendroglioma with 30 samples, each sample with 10,367 genes.	Accuracy.
[81]	DEAP.	EEG of 32 subjects, each having rated 40 music videos of a one-minute duration.	Accuracy.
[23]	ABIDE I.	Dataset includes eyes open rs-fMRI. It considered 251 HC and 201 ASD, all adolescents. Adults, 67 HC and 63 ASD, were also included.	Accuracy.
[84]	ABIDE I.	Dataset in which patients with eyes open during the fMRI session were considered; less than 18 years old; resulting in 251 HC subjects and 201 subjects with ASD.	Accuracy.
[103]	STRING (version 11.0).	Ten proteins were considered to build the protein–protein interaction (PPI) network, which was generated and visualized from the STRING database.	Root-mean-squared error (RMSE).
[71]	HCP.	Task-MRI and rs-MRI acquired from 1200 HC, corresponding to the response to different cognitive tasks.	Accuracy, precision, and recall.
[97]	USC-TIMIT.	rtMRI videos of the upper airway in the mid-sagittal plane and the corresponding speech waveforms of 5 female and 5 male subjects.	Accuracy, precision, false positive, and false negative.
[88]	BCI Competition II; Dataset 1 from BCI Competition IV.	2003 BCI competition dataset EEGs were collected from 1 HC. BCI Competition IV dataset. Continuous EEGs were obtained from 6 HC.	Accuracy and AUC.
[90]	DEAP and synthetic dataset.	Public, peripheral EEG and physiological signal data from 32 participants. Participants watched 40 videos and rated them according to the levels of valence, arousal, liking/disliking, dominance, and familiarity.	Classification accuracy and similarity between the learned graph and the ground truth.
[91]	AMIGOS; ASCERTAIN; DEAP.	The AMIGOS dataset consists of data collected from 40 participants and stores EEG, ECG, and GSR signal data; the ASCERTAIN dataset contains experimentally sourced data from 58 users viewing affective videos, along with EEG, ECG, GSR, and facial activity data; the DEAP dataset has data from 32 participants, and 40 1-min clips of music videos were used as stimuli for the participants.	Accuracy and F1-score.
[25]	iBCIC2020 Competition.	EEG signals from 15 individuals (5 females). The mean age was 31 years, and all subjects were healthy and right-handed.	Accuracy.
[75]	HCP.	100 HC HCP unrelated subjects from the HCP U100 dataset, fMRI acquired with 8 different task conditions (resting state and 7 tasks: emotion, play, language, motor, relationship, social, working memory).	Accuracy.
[85]	Epileptic EEG Data; TSP speech dataset.	For the EEG database, 50 tests of pre-ictal/epileptic ictal EEG signals were carried out. TSP speech is a public dataset, and an utterance of about 2 s duration uttered by a male and a female speaker was considered.	Interference-to-source ratio (ISR), relative graph estimation error (RGEe), AUC, F1, and MD
[95]	Econet-Dynamic; CAMUS.	EchoNet-Dynamic Dataset with 10,030 echocardiography videos; CAMUS dataset contains the medical exams of 500 patients.	Dice coefficient (DC) or F1-score.
[86]	HCP1200 release.	Consists of functional magnetic resonance imaging (fMRI) recordings from 20 HC adult participants. The dataset includes four rs-fMRI recordings, seven task-based fMRI recordings, and one diffusion fMRI recording.	Two measures of temporal complexity: the Hurst exponent and multiscale entropy.
[87]	HCP 1200 Subject Release (S1200).	fMRI data for 302 participants, consisting of 164 females and 138 males (22–35 years, mean = 28.7 ± 3.6). The fMRI data were collected while the participants performed 7 different tasks: emotion, game, working memory, language, relational, social, and motor.	Accuracy, balanced accuracy, F1-scores (macro, micro, and weighted), Matthews correlation coefficient (MCC), precision, and recall.
[99]	HMDB 4.0; CTD; DisGeNET.	The HMDB dataset has 1478 metabolites, 237 diseases, and 3460 known metabolite–disease associations, removing missing and duplicate data. For information on disease-related genes, obtained 3102 genes from the comparative toxicogenomics dataset (CTD) and DisGeNET.	AUC, area under precision–recall (AUPR), F1-score, accuracy, recall, specificity, and precision.
[98]	[144].	It includes data on COVID contamination in the population of the city of Massachusetts from 6 December 2020 to 25 September 2021, for 41 weeks in total, which is collected from the official website.	Anomaly score (a-score).
[101]	circR2Disease.	It considered 431 circRNA-disease associations, which included 365 circRNAs related to 100 diseases from circR2Disease.	Accuracy, precision, recall, F1-score, and AUC.
[104]	Kvasir-SEG; CVC-ClinicDB.	Kvasir-SEG is an open-access dataset of gastrointestinal polyp images, which contains 1000 polyp images; the public and open-access CVC-ClinicDB is composed of 612 image frames extracted from 31 different colonoscopy.	Mean intersection-over-union (mIOU).
[93]	Neurovault; HCP.	Functional MRI signals consisting of 13 subjects with many task experiments and 788 HCP subjects.	Accuracy.
[76]	ADNI.	Total number of 184 subjects in this study. 40 late MCI (LMCI) patients, 77 early MCI (EMCI) patients, and 67 HC.	Accuracy, sensitivity, specificity, F1-score, and AUC.
[92]	Collected for the paper.	It includes 30 patients with inflammatory bowel disease, 13 men and 17 women, mean age (35.3 ± 5.2) years, all right-handed. At the same time, there were 30 HC patients, including 16 males and 14 females, mean age (31.5 ± 2.9) years, all right-handed.	Accuracy, sensitivity, specificity, and F1-score.
[24]	DEAP.	Public dataset with EEG signals from 32 participants when watching 40 60-s video clips. Subjects (50% men and 50% women) were between 19 and 37 years old.	Accuracy.
[94]	Montreal Archive of Sleep Studies (MASS) SS3; SleepEDF.	Full-night polysomnographic recordings. In MASS-SS3, 62 and in SleepEDF 20 healthy individuals were considered	Accuracy, F1-score, and Kappa.
[106]	Collected for the paper.	A total of 79 participants with complete data, with 19 HCs and 60 patients, of which 31 in the cognitive behavioral therapy (CBT) group and 29 in the selective serotonin reuptake inhibitor (SSRI) group.	The statistics are Pearson’s r and *p* values.
[107]	MIT-BIH; Medical Information Mart for Intensive Care (MIMIC); Beth Israel Deaconess Medical Center (BIDMC) PPG and Respiration Dataset; Wrist PPG During Exercise dataset; CAPNOBASE—TBME RR benchmark dataset; Complex System Laboratory (CSL) Pulse Oximetry Artifact Labels.	Datasets with different types of normal and abnormal PPG signal patterns, as well as noisy PPG signals in real time.	Accuracy, processing time, and model size.
[108]	Liver Tumor Segmentation 2017 (LiTS17).	The dataset contains images of 130 patients with a maximum number of CT slices of 623 for each patient. For this study, CT volumes of 10 patients were considered.	Accuracy, Dice coefficient, mean intersection-over-union (IoU), sensitivity, precision, and recall.

## Data Availability

Not applicable.
